# The use of blocking screws with internal lengthening nail and reverse rule of thumb for blocking screws in limb lengthening and deformity correction surgery

**DOI:** 10.1007/s11751-016-0265-3

**Published:** 2016-09-24

**Authors:** Saravanaraja Muthusamy, S. Robert Rozbruch, Austin T. Fragomen

**Affiliations:** 1 Limb Lengthening and Complex Reconstruction Service, Hospital for Special Surgery, 535 East 70th Street, New York, NY 10021 USA; 2 Limb Lengthening and Complex Reconstruction Service, Hospital for Special Surgery, 535 East 70th Street, New York, NY 10021 USA

**Keywords:** Limb lengthening, Deformity correction, Intramedullary lengthening nail, Reverse rule of thumb, Blocking screws

## Abstract

Internal lengthening nail (ILN) is a recent development in limb lengthening and deformity correction specialty. The ILN has the distinct advantage of combining acute deformity correction with gradual lengthening of bone. While using ILN, the short metaphyseal bone fragment may develop a deformity at the time of osteotomy and nail insertion or during bone lengthening because of the wide medullary canal. These deformities are typically predictable, and blocking screws (Poller screws) are helpful in these situations. This manuscript describes the common deformities that occur in femur and tibia with osteotomies at different locations while using ILN in antegrade and retrograde nailing technique. Also, a systematic approach to the appropriate use of blocking screws in these deformities is described. In addition, the “reverse rule of thumb” is introduced as a quick reference to determine the ideal location(s) and number of blocking screws. These principles are applicable to limb lengthening and deformity correction as well as fracture fixation using intramedullary nails.

## Introduction

Limb lengthening and deformity correction is a rapidly evolving orthopedic subspecialty that is gaining international popularity. Part of the reason for this peaked interest is the trend towards internal fixation instead of total reliance on external fixation. Most notably, the intramedullary nail (IMN) has taken the center stage for its ability to acutely correct deformities and/or gradually lengthen both the femur and the tibia [1]. The emergence of a reliable internal lengthening nail (ILN) has added the distinct advantage of combining acute deformity correction with gradual lengthening of bone. The ILN frequently does not provide adequate stability in metaphyseal regions having wide medullary canals [1, 2]. Intramedullary nailing after corrective osteotomy requires that the deformity be corrected before reaming and nail insertion. If the deformity is not corrected and reaming ensues, the IM nail will follow the path of the reamer making the correction impossible. Similarly, during bone lengthening with an ILN, the bone may angulate and result in malalignment and deformity. These deformities are mostly predictable depending on the location of the osteotomy. Pivotal to the success of the ILN is the appropriate use of blocking screws. Well-placed blocking screws will aide in the reduction of a deformity, hold an osteotomy reduction in place especially during the lengthening process, and will help prevent novel distraction-induced malalignment of the bone fragments [3, 4]. But deciding the location(s) and the number of blocking screws is often difficult and confusing. The aim of this manuscript is to present a detailed review of the common patterns of deformities that occur during limb lengthening and deformity correction using ILN, to present a systematic approach to the appropriate use of blocking screws and to introduce the “reverse rule of thumb” as a quick reference for surgeons to know the ideal location(s) and the number of blocking screws. The “reverse rule of thumb” for blocking screws can be applied to any long bone, any fragment (proximal or distal), and any nailing technique (antegrade or retrograde as well as open or closed nailing). The patterns of deformity and the reverse rule of thumb are relevant to limb lengthening, deformity correction and fracture fixation using interlocked intramedullary nails.

## Patterns of deformity (angulation)

The commonly encountered patterns of deformity are bone specific and osteotomy site specific. Deformities can be preexisting or can arise as the result of distraction osteogenesis. Preexisting deformities are corrected acutely. Deformities of the proximal femur meta-diaphysis are best controlled with an intraoperative external fixator and a fixator-assisted nailing technique [5]. Once the nail is in place, it does not require blocking screws in the proximal fragment. The distal fragment could possibly go into varus or valgus during lengthening and may benefit blocking screws. This pattern of lengthening-induced deformity is not common. Deformities of the distal femur metaphysis are best corrected by first placing the blocking screw, then reaming, and then passing the nail. Additional screws can be added as needed. Deformities of the proximal tibial metaphysis are treated in the same way with insertion of a directional blocking screw to guide the trajectory of the reamer ensuring a proper correction of the malalignment. Diaphyseal deformities may not require blocking screws. The nail tends to correct these deformities naturally. If the bone quality is deemed poor, then blocking screws may help prevent deformity.

Deformity can occur in the coronal (varus/valgus), sagittal (procurvatum/recurvatum), and axial (rotation/length) planes. All of these deformities, except the length, can be corrected acutely during nail insertion. Lengthening-induced deformity assumes familiar patterns similar to those seen with external fixator-assisted lengthening. Proximal femoral osteotomy and antegrade nailing induce varus at the osteotomy site, especially while using thinner nails and a trochanteric entry point. Distal femoral osteotomy with retrograde nailing consistently creates flexion (procurvatum) at the osteotomy site, but induction of varus or valgus is less predictable. A thinner diameter ILN may tend to bend into varus, and a stiffer nail will create lateral mechanical axis deviation due to lengthening along the anatomical axis. Lengthening through a proximal tibial osteotomy predictably creates a procurvatum deformity. The bone will often deform into valgus as well, but if the starting point of the nail is slightly lateral to center, then the osteotomy may deform into varus. Therefore, lengthening-induced coronal plane deformity is highly dependent on the positioning of the nail in the proximal tibial fragment (Table 1).

**Table 1:** Predicted deformities and the ideal location(s) and number of blocking screws while lengthening the femur and tibia using internal lengthening nail with osteotomies at different locations

Osteotomy site	Nailing technique	Predicted deformity	Blocking screw(s) in each bone fragment near the osteotomy
Proximal Femur	Antegrade	Varus	2 Medial screws
		Valgus	2 Lateral screws
		Procurvatum	1–2 Posterior screw(s)
Mid Femur	Antegrade or retrograde	Varus	2 Medial screws
		Valgus	2 Lateral screws
		Procurvatum	1–2 Posterior screw(s)
Distal Femur	Retrograde	Varus	2 Medial screws
		Valgus	2 Lateral screws
		Procurvatum	1–2 Posterior screw(s)
Proximal Tibia	Antegrade	Varus	2 Medial screws
		Valgus	2 lateral screws
		Procurvatum	1–2 Posterior screw(s)
Mid Tibia	Antegrade	Varus	2 Medial screws
		Valgus	2 Lateral screws
		Procurvatum	1–2 Posterior screw(s)

We did not encounter a recurvatum deformity of the femur or tibia, and a distal tibial osteotomy was unnecessary in our experience

Femoral lengthening along the anatomical axis using an ILN leads to lateral mechanical axis deviation, but tibial lengthening along the anatomical axis does not change the mechanical axis since they are parallel

## Technique for blocking screws

For the purpose of inserting blocking screws, the proximal and distal fragments have to be considered individually. Factors contributing to the stability of bone–nail construct are stability at the nail entry site, a snugly fitting medullary canal, impaction of the nail tip into the metaphysis and the interlocking screws. When the osteotomy is done in the metaphysis, the nail is usually centered in the longer bone fragment and may not need blocking screws unless the IM canal is significantly wider than the nail at the osteotomy site. Screws can still be used in the longer fragment if stability is a concern. The shorter metaphyseal fragment has a wide medullary canal and is at risk of toggling around the nail and developing undesirable angulation. Accordingly, the shorter fragment frequently needs blocking screws for additional stability.

Deformities in coronal and sagittal planes should be considered separately. In varus or valgus deformity, the blocking screws should be inserted in the anteroposterior plane in each fragment. Similarly, in procurvatum or recurvatum deformity, the blocking screws should be inserted in the mediolateral plane in each fragment. If the deformity is in oblique plane, it could be stabilized by blocking screws placed perpendicular to the plane of maximum deformity or blocking screws placed in both coronal and sagittal planes.

The ILN is a titanium nail, and therefore, titanium blocking screws should be used. The screws need to be strong enough to resist the reamer and control the bone fragment. We use 5-mm, fully threaded, IMN interlocking screws from any manufacturer. The position of the screws is planned preoperatively and reproduced in the operating room. The screws are inserted under fluoroscopy using free-hand technique. Blocking screws placed too close to the nail can contact the nail. This is not a problem if the screws are in the non-moving fragment. If the screws are in the moving fragment (distal femur with an antegrade nail, proximal femur with a retrograde nail, and distal tibia with antegrade nail), they should not be placed too close to the nail. A blocking screw that impinges on the nail as the nail tries to slide in the bone could produce too much resistance to lengthening and may jeopardize the distraction process. Therefore, we suggest 1–2 mm space between the blocking screw and the nail.

## Deciding the location(s) of blocking screws

**Fig. 1:**
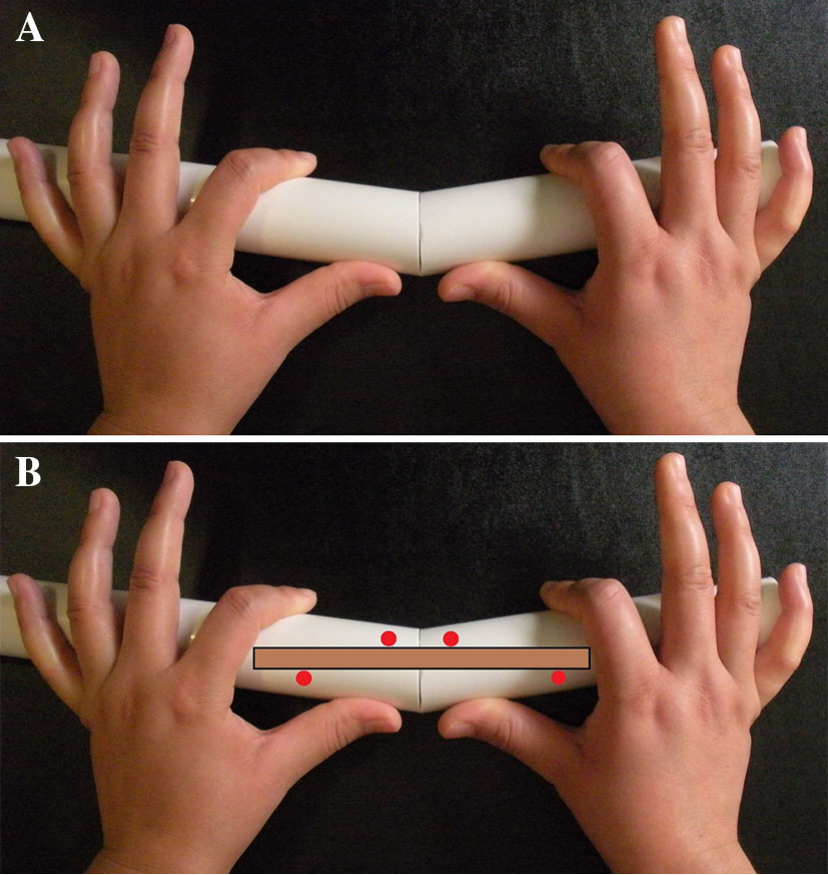
**a** Holding the bone with the thumbs and index fingers of both hands as if you are manually trying to correct the deformity. The thumbs of both hands are placed on the convex side of the deformity near the apex or osteotomy site, and the index fingers are placed away from the apex or osteotomy site on the concave side. **b** The *gray bar* with *black outline* represents nail. The *red circles* indicate the locations where the blocking screws should be inserted. They are inserted adjacent to the nail on the side that is OPPOSITE to where the thumbs and index fingers are placed on the bone

The deformity may exist before the osteotomy or may appear immediately after nail insertion following an osteotomy. It may also arise postoperatively due to displacement of bone fragments or distraction. To decide the locations of screws, the surgeon should know the plane of existing deformity or be able to speculate the expected deformity. To decide the ideal locations of the blocking screws, the “reverse rule of thumb” is helpful. This technique involves three steps: (1) assess or speculate the deformity: understand the direction of existing deformity that will be corrected with nailing or speculate the deformity that could occur later during lengthening; (2) manually correct the deformity: envision trying to manually correct the deformity by holding the bone with both hands. The thumbs of both hands are placed on the convex side of the deformity near the apex, and the index fingers are placed away from the deformity on the concave side. (3) Insert the blocking screws on the side of the nail OPPOSITE to where the thumbs and index fingers are placed on the bone (Fig. 1). The blocking screw abuts the intramedullary nail preventing unwanted movement of the bone around the nail.

## Deciding the number of blocking screws

**Fig. 2:**
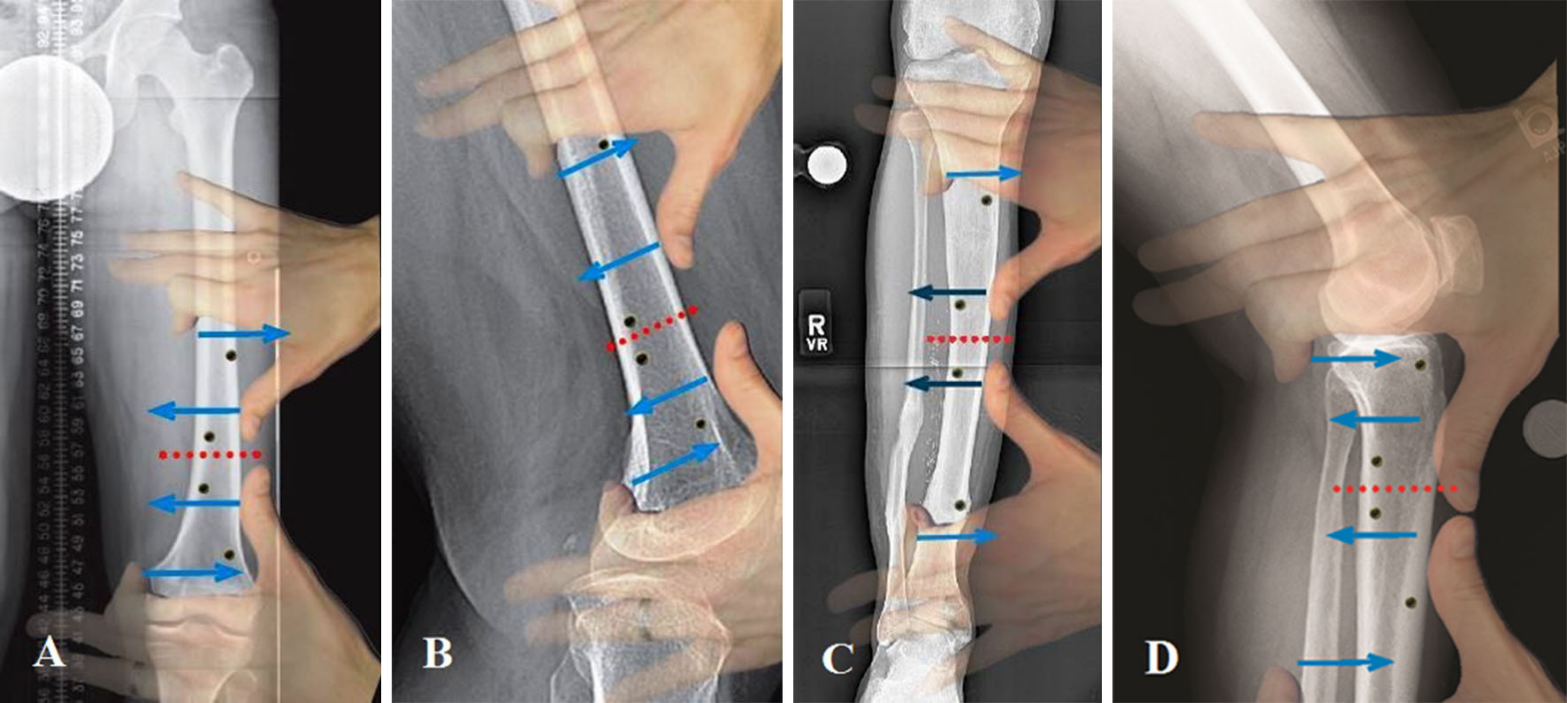
**a** Correction of distal femur varus deformity: The thumbs of both hands are placed laterally over the apex of the deformity, and the index fingers are placed on the concave side away from the apex. The *red dotted line* indicates the osteotomy site, and the *blue arrows* indicate the direction of force to correct the deformity. The *black dots* indicate the locations where the blocking screws should be inserted using the reverse rule of thumb. They are inserted on the side that is OPPOSITE to where the thumbs and index fingers are placed on the bone. **b** Correction of distal femur procurvatum deformity: The bone is not deformed, but the distal fragment is expected develop procurvatum deformity during lengthening. The thumbs of both hands are placed anteriorly where the apex of the procurvatum deformity would lie. The *black dots* indicate the locations where the blocking screws should be inserted. **c** Correction of mid tibia valgus deformity: The *black dots* indicate the locations of blocking screws. **d** Correction of proximal tibia procurvatum deformity: The bone is not deformed, but the proximal fragment is expected develop procurvatum deformity during lengthening. The *black dots* indicate the locations of blocking screws

If the nail is not centered in the bone fragment, either near the osteotomy site or away from the osteotomy site, then only one blocking screw is used at the displaced end of the bone fragment. If both ends are not centered over the nail, two blocking screws are used, one at each end of the bone fragment. While using two blocking screws in one fragment, it is better to insert one screw close to the osteotomy site and the second screw at the other end of the fragment to maximize the stability. Each screw location suggested by reverse rule of thumb need not be utilized. They are utilized only as needed depending on whether one or both ends of each bone fragment are deformed (Fig. 2).

### Specific case scenarios

The following is a description of different scenarios specific to the bone, location of osteotomy, and the type of nailing (Table 1).

### Femur: proximal osteotomy and antegrade nail

**Fig. 3:**
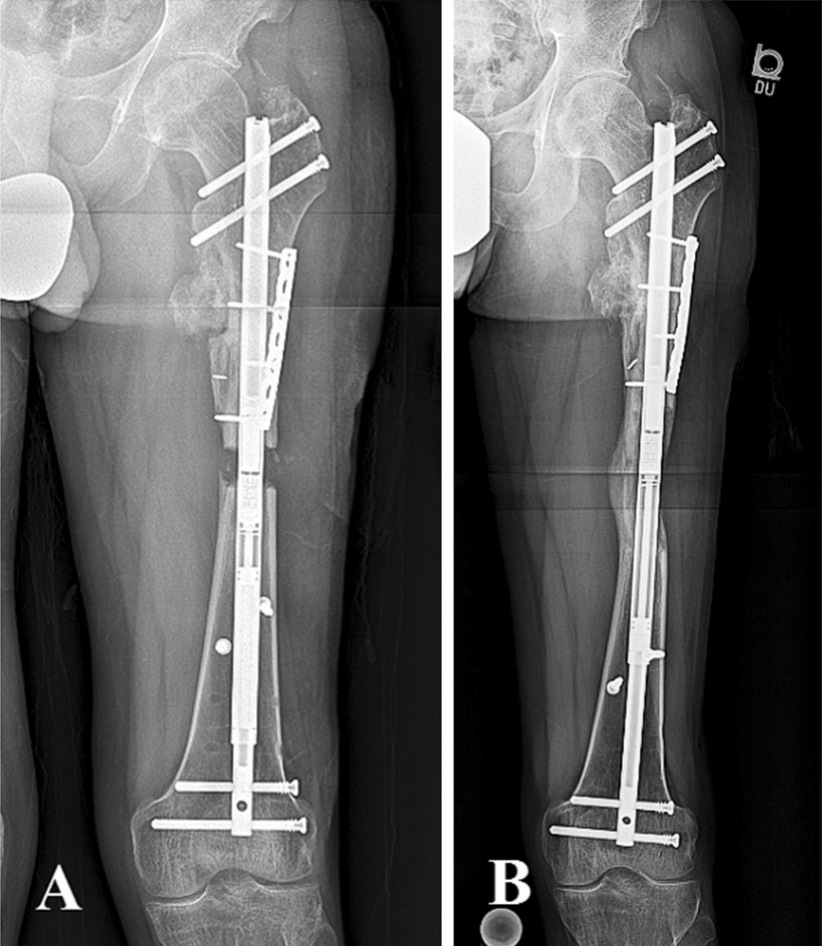
Antegrade femoral nail. **a**, **b** Represent radiographs taken at early distraction and consolidation phases, respectively. Blocking screws were not needed in the proximal fragment. One medial screw and one lateral screw were inserted in the distal fragment near the osteotomy site to prevent varus or valgus tilt of the fragment

The need for blocking screws in the proximal femur is left to the discretion of the surgeon. However, we have never found the need for them. The use of blocking screws in the distal femur can be beneficial in preventing the nail from sliding over the smooth distal locking screws during lengthening (Fig. 3). Comminution at the osteotomy site and poor bone quality may warrant blocking screws. The screws can be placed both medially and laterally to the nail since the nail/bone could shift into either varus or valgus. Sagittal plane deformity is extremely unlikely.

### Femur: distal osteotomy and retrograde nail

**Fig. 4:**
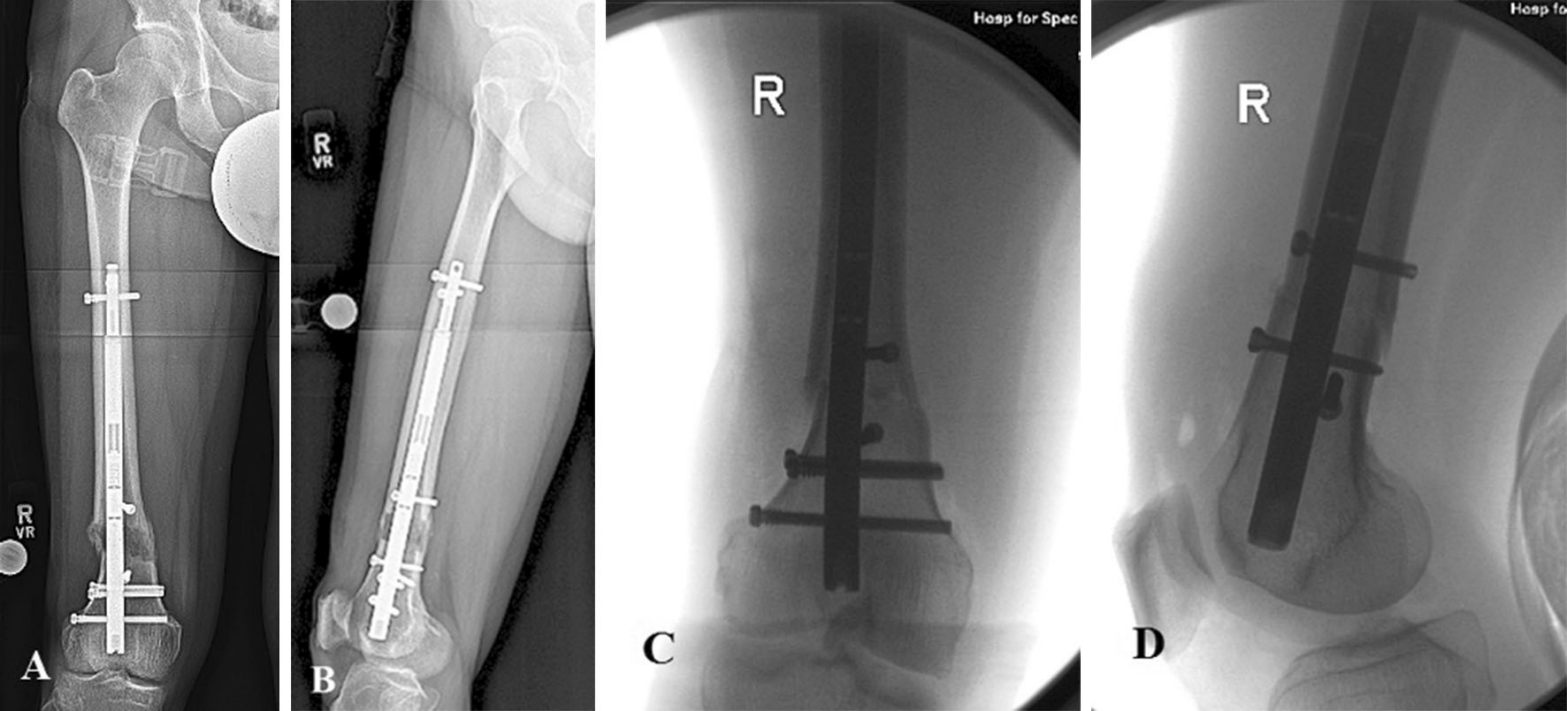
Retrograde femoral nail with distal femoral osteotomy. Anteroposterior radiograph (**a**) and intraoperative fluoroscopic anteroposterior view (**b**) show two medial blocking screws, one in proximal fragment and one in the distal fragment near the osteotomy site used to prevent varus angulation of the proximal and distal fragments, respectively. Lateral radiograph (**c**) and the intraoperative fluoroscopic lateral view (**d**) show one posterior blocking screw in the distal fragment near the osteotomy site used to prevent procurvatum deformity

This is the most common scenario that relies on blocking screws for success. A retrograde nail is indicated for the correction of existing coronal plane deformity. Either one or two blocking screw(s) are used to correct and maintain the new alignment. For a varus deformity, blocking screws are positioned medial to the nail. Lateral screws are used for valgus deformity. Sagittal plane deformity does not typically present preoperatively but is the most common complication of femur lengthening. One blocking screw placed posterior to the nail in the distal fragment is critical. A second similar screw can be placed in the proximal fragment close to the osteotomy site (Fig. 4). Technically, the reverse rule of thumb suggests additional blocking screws far from the osteotomy site, closer to the joints. From a practical perspective, these screws are rarely needed. The far ends of the nail are well secured in the bone. The metaphyseal bone near the osteotomy is least controlled by the nail and needs blocking screws.

### Tibia: proximal osteotomy and antegrade nail

**Fig. 5:**
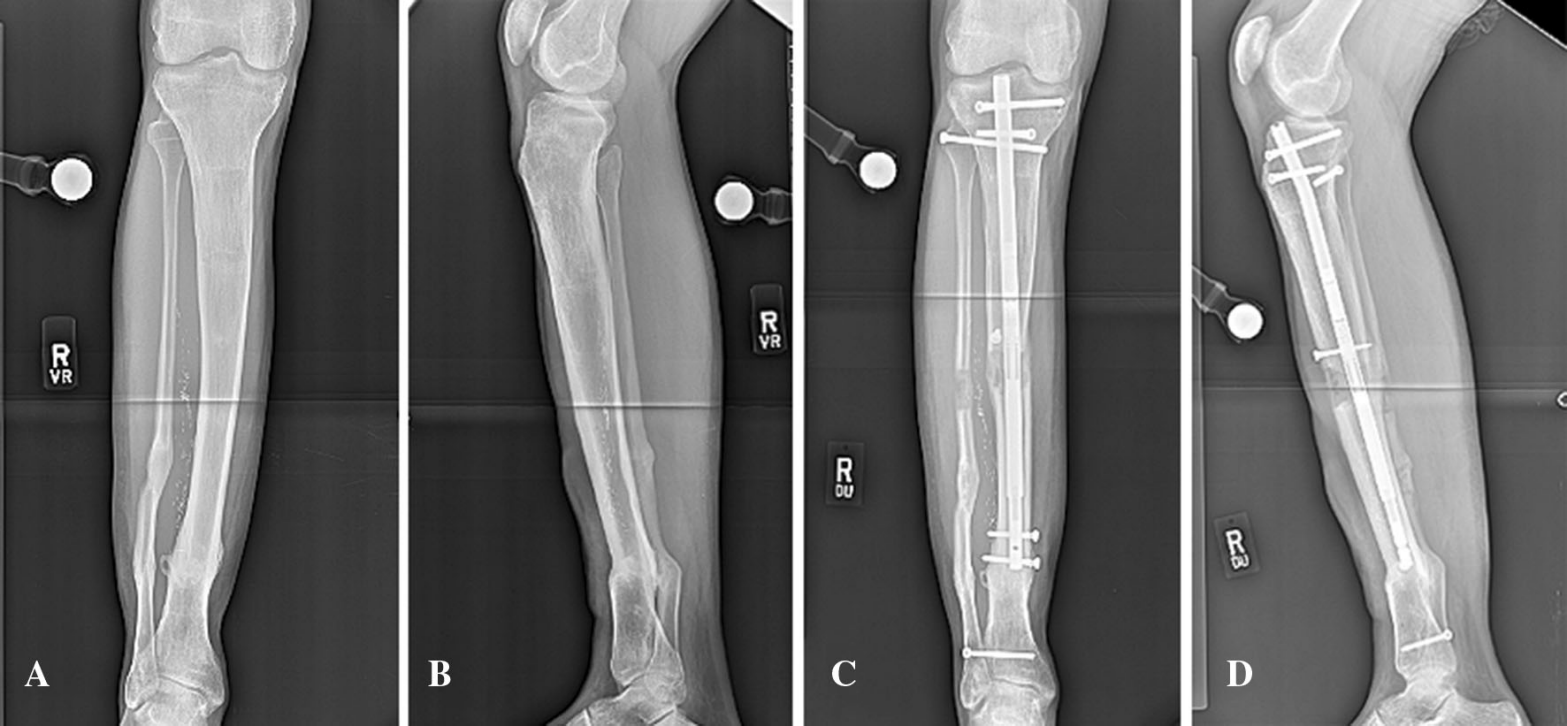
Antegrade tibial nail with mid-tibial osteotomy. Preoperative anteroposterior (**a**) and lateral (**b**) radiographs show preexisting valgus deformity of mid tibia. One lateral blocking screw was inserted near the osteotomy site in the proximal fragment to avoid valgus deformity during lengthening. Also, a blocking screw was inserted posterior to the nail in the proximal fragment before the reaming to guide the nail. Radiographs (**c**, **d**) represent post operative anteroposterior and lateral views at the end of distraction, respectively

Commonly, IM nails are used to correct proximal tibial deformity. Varus angulation requires a blocking screw medial to the IM nail in the proximal fragment and often in the distal fragment close to the osteotomy site. A valgus tibial deformity is treated with a screw placed lateral to the IM nail on either side of the osteotomy. Often, these screws are inserted before reaming to ensure a correction of the deformity. This configuration is highly susceptible to angulation during lengthening. A blocking screw must also be placed posterior to the IM nail in the proximal fragment, preferably before the reaming (Fig. 5).

## Discussion

Gerhard Küntscher is credited with the invention of intramedullary nail fixation in 1939 for femur fractures. The early nails were unlocked, and they failed to maintain length, rotation, and angulation. To overcome these problems, Modny and Bambara introduced locked intramedullary nails [6]. These locked nails were able to control length and rotation especially in the mid-diaphyseal region. But, they were not very effective in controlling angulation of the shorter bone fragment because of the wide medullary canal in metaphyseal region. Use of opposing interlocking screws in oblique plane and biplanar interlocking screws (both in coronal and sagittal planes) have been advocated to increase the stability in such situations [7–9]. Another approach is the use of blocking screws (also called Poller screws) with the interlocking nail to increase the stability.

In 1999, Krettek et al. [3, 4] introduced the use of blocking screws. In this technique, screws are inserted through the medullary canal immediately adjacent to the nail to prevent relative motion of the bone and the nail. In a cadaveric study, Krettek et al. [3] described the use of blocking screws in coronal and sagittal planes with the help of a custom-made jig. They showed that in proximal and distal tibial fractures treated with small-diameter intramedullary nails, the blocking screws increase the stability and prevent malalignment and/or instability. They recommended placing screws on the concave side of the deformity, one proximally and one distally. Krettek et al. [4] reported that with the use of Poller screws (blocking screws) with small-diameter IM nails in metaphyseal tibial fractures for supplemental stability, the mean loss of reduction was 0.5° in the frontal plane and 0.4° in the sagittal plane. Similarly, various authors have described the use of interlocking intramedullary nail in combination with blocking screws in metaphysis of long bones to improve reduction, prevent secondary displacement, and to augment and maintain the alignment and stability [10–14]. The use of blocking screws has also been reported to hasten the bone healing in fractures [15] and nonunions [16] as well as to reduce the risk of implant failure [17]. Stedtfeld et al. [18] indicated that the blocking screws around the nail relieves axial strain in the fixation construct and called these screws “transmedullary support screws.” The use of blocking screws before reaming and insertion of nail in the segment of bone where the medullary cavity is wide to guide the trajectory of reamer and nail in the right direction and to aid in reduction has been described [11, 19, 20]. Also, with interlocked intramedullary nailing using one or two “parallel” locking screws, translation of the bone fragment over the interlocking screw has been described [7–9].

The number and the locations of the blocking screws are crucial to the successful outcome. Deciding the location(s) and the number of blocking screws is often difficult and confusing. Krettek et al. [3, 4] recommended placing one screw proximally and one distally on the concave side of the deformity. Hannah et al. [21] described placing the blocking screw in the acute angles formed between the long axis of the bone segment and the fracture plane in oblique fractures. Stedtfeld et al. [22] described different clinical scenarios with fractures involving the proximal and distal metaphysis of long bones and improving the stability with the use of one or two blocking screws. Seyhan et al. [23] suggested that the blocking screws must be inserted 1–3 cm away from the fracture line to avoid propagation of the fracture.

The intramedullary lengthening nail (ILN) can be used for lengthening alone or deformity correction followed by lengthening. Similarly, interlocked intramedullary nail (IMN) can be used in bone lengthening (lengthening over nail technique, LON) as well as for fixation after deformity correction. These nails (ILN and IMN) frequently do not provide adequate stability in metaphyseal regions having wide medullary canals [1, 2]. Intramedullary nailing after corrective osteotomy requires that the deformity be corrected before reaming and nail insertion. If the deformity is not corrected and reaming ensues, the IM nail will follow the path of the reamer making the correction impossible. Similarly, during bone lengthening with an ILN, the bone fragments may be well aligned after the index surgery, but a deformity may develop during lengthening. This problem is further compounded by the need for over-reaming and the use of undersized nails which is common in lengthening. Knowledge of the common patterns of deformity and the reverse rule of thumb is extremely helpful for ensuring the proper use of blocking screws to mitigate this complication.

## Conclusion

The knowledge of the common patterns of deformity associated with intramedullary nailing and the “reverse rule of thumb” help in deciding the location(s) and the number of blocking screw(s). These principles are applicable to bone lengthening, deformity correction, and fracture fixation using interlocked intramedullary nails.
